# How Does Pulmonary Function Change in Patients With Severe Thoracic Scoliosis 2 Years After One‐Stage Low‐Grade Osteotomy and Posterior Corrective Surgery?

**DOI:** 10.1111/os.70236

**Published:** 2025-12-29

**Authors:** Junduo Zhao, Yang Jiao, Yizhen Huang, Heng Sun, Haoyu Cai, Haojie Chen, Jianxiong Shen

**Affiliations:** ^1^ Department of Orthopedics Peking Union Medical College Hospital, Peking Union Medical College, Chinese Academy of Medical Science Beijing China; ^2^ Department of Surgery, Department of Breast Surgery Peking Union Medical College Hospital, Peking Union Medical College, Chinese Academy of Medical Science Beijing China

**Keywords:** apical correction and global balance, pulmonary function, scoliosis, surgery

## Abstract

**Objective:**

Severe scoliosis is often accompanied by moderate‐to‐severe pulmonary dysfunction. Numerous surgical methods are available for the treatment of severe scoliosis, but the effect of each method on postoperative pulmonary function (PF) remains controversial. Apical region correction and global balance (ACGB) is an effective surgical strategy to treat severe scoliosis, using Schwab I–II osteotomies and simple one‐stage surgery. Herein, we explore the effect of the ACGB surgical strategy on postoperative PF values in patients with severe scoliosis at 2‐year follow‐up.

**Methods:**

Patients who underwent ACGB for scoliosis between 2015 and 2020 were enrolled, PF and radiological outcomes were evaluated preoperatively and at 2‐year follow‐up. PF values included forced expiratory volume in 1 s (FEV1), forced vital capacity (FVC), and percent‐predicted values (FVC% and FEV1%). Paired *t*‐test, Pearson and Spearman correlation analyses, and multiple linear regression were used to analyze changes in PF values and associated factors.

**Results:**

In total, 36 patients (12 male and 24 female; mean age, 20.1 years) who underwent ACGB surgery were enrolled. Preoperative PF values showed restrictive ventilatory dysfunction. At 2‐year follow‐up, the PF values showed significant improvements. FVC, FEV1, FVC%, and FEV1% showed mean increases of 0.72, 0.68 L, 10.3%, and 9.8%, respectively. Changes in PF values were significantly correlated with age, thoracic height, preoperative FVC%, and preoperative FEV1%.

**Conclusion:**

ACGB significantly improves PF in patients with severe scoliosis at 2‐year follow‐up. The increased thoracic height may be crucial for improving PF values, while patients with poorer preoperative PF may show greater postoperative improvement.

## Graphical abstract

One‐stage low‐grade osteotomy surgery with apical region correction and global balance significantly improved pulmonary function in severe thoracic scoliosis patients at 2‐year follow‐up. Increased thoracic height is regarded as the key correlating factor.

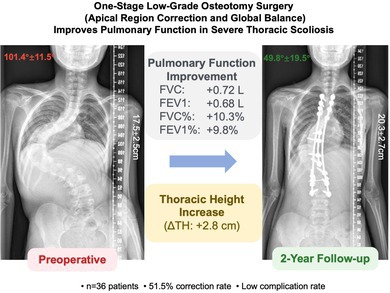

**Abbreviations**
ACGB: apical region correction and global balance
FEV1: forced expiratory volume in 1 s
FEV1%: percent forced expiratory volume in 1 s
FVC: forced vital capacity
FVC%: percent forced vital capacity
PF: pulmonary function
RVD: restrictive ventilatory dysfunction
SVA: sagittal vertical axis
TD: thoracic depth
TH: thoracic height
TK: thoracic kyphosis
TS: trunk shift
TW: thoracic width
VCR: vertebral column resection

## 1 Introduction

Scoliosis is defined as a three‐dimensional deformity of the spine that can lead to distortion of the chest wall, reduce lung volume, and often results in restrictive ventilatory dysfunction (RVD) [1, 2]. Severe scoliosis is defined as a main curve exceeding 90° [3]. Patients with severe scoliosis often present with moderate‐to‐severe RVD or pulmonary function (PF) deficits, which lead to irreversible PF impairment and respiratory failure if left untreated [4, 5]. Maintaining or improving PF is considered a primary goal of surgical treatment for patients with severe scoliosis. Although several surgical methods, such as high‐grade osteotomy, anterior release compared with posterior spinal fusion, and surgical correction after traction, have shown to be useful for the treatment of severe scoliosis, the effect of each method on postoperative PF remains controversial [6, 7, 8].

Apical region correction and global balance (ACGB) is a one‐stage posterior‐only surgical strategy to treat severe scoliosis and can achieve a reasonable corrective effect with a relatively low incidence of complications [9]. However, the effect of ACGB on postoperative PF in patients with severe scoliosis has not yet been reported. In this study, we aimed to: (i) investigate the impact of the ACGB surgical strategy on postoperative PF at 2‐year follow‐up in patients with severe thoracic scoliosis; (ii) identify the possible impact variables related to changes in PF, with particular focus on thoracic dimensional changes; and (iii) evaluate the advantages of this low‐grade osteotomy approach in preserving and improving PF compared to high‐grade osteotomy techniques. This study provides evidence for a safer surgical alternative that achieves both satisfactory deformity correction and PF improvement, with potential implications for future treatment strategies in severe scoliosis management.

## 2 Methods

### 2.1 Patients

This clinical study was conducted from September 2015 to August 2022 at our hospital. Overall, 36 patients (24 female and 12 male) with severe scoliosis who required surgery were enrolled in the study. The inclusion criteria were: (1) the major curve of scoliosis was thoracic curve (the apex was located on T2–T11/12 disc); (2) Cobb angle greater than 90°; (3) angle of kyphosis less than 100°; (4) no previous traction treatment, spinal surgery, or lung surgery; and (5) no other diseases affecting respiratory function. The exclusion criteria were: (1) patients with primary pulmonary diseases such as chronic obstructive pulmonary disease, asthma, or interstitial lung disease; (2) patients with cardiac dysfunction or congenital heart disease affecting respiratory function; (3) patients with chest wall deformities independent of scoliosis; (4) patients with incomplete radiological or PF data; (5) patients lost to follow‐up before 2 years postoperatively; and (6) patients who declined to participate in the study.

All patients with severe thoracic scoliosis underwent ACGB. PF tests were performed preoperatively and 2 years postoperatively. All surgeries were performed by a senior spinal surgeon at our hospital. Preoperative data, including age, diagnosis, weight, and height, were obtained from medical records, while postoperative follow‐up time, height, weight, and complications were obtained from outpatient medical records.

### 2.2 Surgical Related Data

During surgery, continuous spinal cord monitoring was performed under general intravenous anesthesia. In the fusion range, bilateral facet joints were partially or fully resected (Schwab grade I or II osteotomy). Three major steps were involved in the surgical procedure after pedicle screw implantation: (1) apical region correction, (2) global balance from concave side, and (3) convex side support [9]. Surgical‐related data, including operation time, intraoperative blood loss, range of operated vertebrae, number of implanted pedicle screws, number of operated thoracic vertebrae, and perioperative complications, were reviewed for all patients.

### 2.3 Radiological Measurements

All patients underwent anteroposterior and lateral radiography of the whole spine preoperatively, postoperatively, and during the regular follow‐ups (February 2015 to September 2022). Radiographic measurements were independently performed by two attending orthopedic surgeons. Spinal and thoracic dimension parameters were collected before the operation and at the 2‐year follow‐up. The relevant spinal parameters included: (1) Cobb's angle, (2) flexibility of the main curve, (3) trunk shift (TS), (4) thoracic kyphosis (TK), and (5) sagittal vertical axis (SVA).

In this study, kyphosis was divided into three types according to TK: hypokyphosis, normal kyphosis, and hyperkyphosis. Hypokyphosis was defined as TK < 20°, normal kyphosis as TK between 20° and 45°, and hyperkyphosis as TK > 45° [10]. The thoracic dimensions parameters were as follows: (1) thoracic height (TH), defined as the vertical distance between the centers of T1 and T12 in the posteroanterior view; (2) thoracic width (TW), defined according to Obikili's measurement [11] as the transverse distance between both sides of internal surfaces of ribs and above the diaphragm; and (3) thoracic depth (TD), defined as the distance between the chest wall and the apical vertebrae's anterior margin in the lateral view.

### 2.4 PF Test

The patients completed PF tests preoperatively and at the 2‐year follow‐up. PF tests were consistently performed using a standard ultrasound spirometer (MasterScreen; CareFusion) in the sitting position to ensure consistency and reproducibility. During each evaluation, spirometry was repeated three times, and the highest value was obtained. Forced vital capacity (FVC), forced expiratory volume in 1 s (FEV1), percent predicted values (FVC%, FEV1%), and the FEV1/FVC ratio were calculated. RVD was defined as an FVC% < 80%. Classification of RVD severity was based on FEV1% and included mild (FEV1% ≥ 70%), moderate (FEV1% 50%–69%), and severe (FEV1% < 50%) [12]. Considering the true height of patients with severe scoliosis could not be measured, FVC% and FEV1% were calculated based on arm span. The difference between the PF values at the 2‐year follow‐up and the corresponding preoperative PF values was calculated as the improvement in PF.

### 2.5 Statistical Analysis

Radiological and PF values were compared between the two time points (before surgery and 2 years after surgery) using the paired *t*‐test. Normally distributed variables were assessed using Pearson's correlations. Correlations between the changes in PF values and the impact of all variables, including basic data, surgery‐related data, and radiographic parameters were analyzed. Categorical data were analyzed using Spearman's correlation, and continuous data were analyzed using Pearson's correlations. Variables with a significant linear relationship with improvements in PF values were selected. Multiple linear regression analysis (stepwise method) was used to analyze the relationship between the selected variables and improvements in PF values. The mean ± standard deviation was used to express the measurement data. Utilizing analysis of variance, a comparison was made among the three RVD subgroups (mild vs. moderate vs. severe) for PF values. SPSS statistical software (version 25.0; IBM, NY, USA) was used for statistical analysis. *p* < 0.05 was considered statistically significant.

## 3 Results

### 3.1 Surgical and Radiographic Outcomes

A total of 36 patients were included in this study, including 24 females and 12 males with a mean age of 20.1 years (range, 11–49 years). The mean body mass index (BMI) was 19.15 ± 3.6 kg/m^2^. Scoliosis was diagnosed as congenital in 14 cases, idiopathic in 12, neuromuscular (syringomyelia‐associated scoliosis) in 6, and Marfan syndrome in 4. The mean operation duration and intraoperative blood loss were 292.5 ± 47.6 min and 920 ± 332 mL, respectively. The mean number of fusion segments was 13.5 ± 1.2. To address potential confounding effects of etiological heterogeneity, subgroup analysis among the four etiological groups was performed. Baseline characteristics including age, gender, preoperative major curve Cobb angle, thoracic kyphosis, and curve flexibility showed no significant differences among groups (all *p* > 0.05). Also, postoperative PF improvements at 2‐year follow‐up and surgical correction outcomes had no significant differences among groups (all *p* > 0.05).

Overall, the mean preoperative major curve of 101.4° ± 11.5°was significantly corrected to 49.8° ± 19.5° at the 2‐year follow‐up, showing a 51.5% mean correction rate. The postoperative kyphosis correction rate was 28.8%; preoperative flexibility was 19.1% ± 8.3%; and TH significantly improved from 17.5 ± 2.5 to 20.3 ± 2.7 cm. There were no significant differences in TW and TD. Details of the radiographic outcomes are listed in Table 1.

**TABLE 1:** Comparison of radiographic outcomes between 2‐year follow‐up and preoperative imaging.

Radiological outcomes	Preoperative	2‐year follow‐up	CR^a^ (%)	*p*
Major curve (°)	101.4 ± 11.5	49.8 ± 19.5	51.5	0.000*
Thoracic kyphosis (°)	59.1 ± 21.6	42.1 ± 12.6	28.8	0.018*
Hypokyphosis (*n* = 3)	10.8 ± 6.1	26.5 ± 4.9		0.32
Normal kyphosis (*n* = 9)	42.5 ± 4.6	40.2 ± 6.2		0.18
Hyperkyphosis (*n* = 24)	71.4 ± 8.3	44.8 ± 10.8		0.001*
Trunk shift (mm)	27.8 ± 14.3	17.1 ± 13.7	/	0.14
Sagittal vertical axis (mm)	36.1 ± 22.6	25.5 ± 13.8	/	0.26
Thoracic height (cm)	17.5 ± 2.5	20.3 ± 2.7		0.002*
Thoracic width (cm)	21.6 ± 2.0	21.2 ± 1.9		0.29
Thoracic depth (cm)	12.4 ± 3.6	11.9 ± 3.0	/	0.35

^a^ CR indicates the correction rate.

* Indicates a significant difference (*p* < 0.05).

### 3.2 PF Parameters Improve 2 Years After ACGB

**FIGURE 1:**
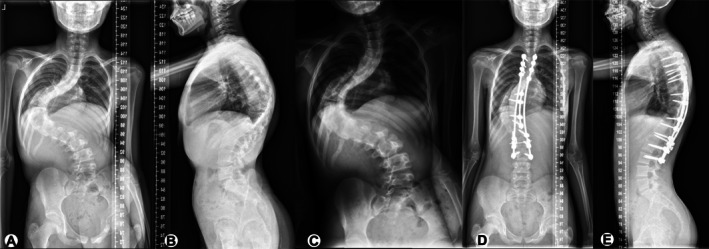
Representative case 1. Example case of a 12‐year‐old female patient with severe and rigid neuromuscular scoliosis. The preoperative Cobb angle of the main curve was 118°, the thoracic height was 16.9 cm (A), kyphosis was 82° (B), and the major curve in bending film was 110° (C). Preoperative pulmonary function indicated restrictive ventilatory dysfunction (FVC = 1.52 L, FEV1 = 1.40 L, FVC% = 55.5%, and FEV1% = 58.9%). She underwent apical region correction and global balance surgery from the T3 to L3. After the 2‐year follow‐up, scoliosis and kyphosis were corrected to 33° (D) and 51° (E), respectively. Her postoperative thoracic height was 21.1 cm (D). Pulmonary function test results improved as follows: FVC = 2.37 L, FEV1 = 2.14 L, FVC% = 74.6%, and FEV1% = 75.5%. FEV1, forced expiratory volume in 1 s; FVC, forced vital capacity; FVC% and FEV1%, percent predicted values.

**FIGURE 2:**
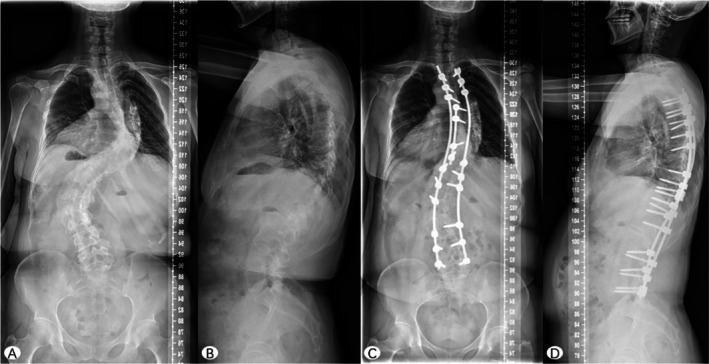
Representative case 2. An example case of a 49‐year‐old female patient with severe and rigid adult scoliosis. The preoperative Cobb angle of the thoracic curve and lumbar curve was 93° and 87°, respectively (A). The preoperative thoracic height was 20.8 cm (A), and the thoracic depth was 12.2 cm (B). Preoperative pulmonary function indicated restrictive ventilatory dysfunction (FVC = 2.21 L, FEV1 = 1.90 L, FVC% = 65.8%, and FEV1% = 66.4%). She underwent apical region correction and global balance surgery from T3 to L5. At the 2‐year follow‐up, the thoracic curve and lumbar curve were corrected to 45° and 41° (C), respectively. Her postoperative thoracic height and depth were 23.7 cm (C) and 11.8 cm (D). Pulmonary function test results were improved as follows: FVC = 2.55 L, FEV1 = 2.25 L, FVC% = 69.1%, and FEV1% = 70.6%. FEV1, forced expiratory volume in 1 s; FVC, forced vital capacity; FVC% and FEV1%, percent predicted values.

**FIGURE 3:**
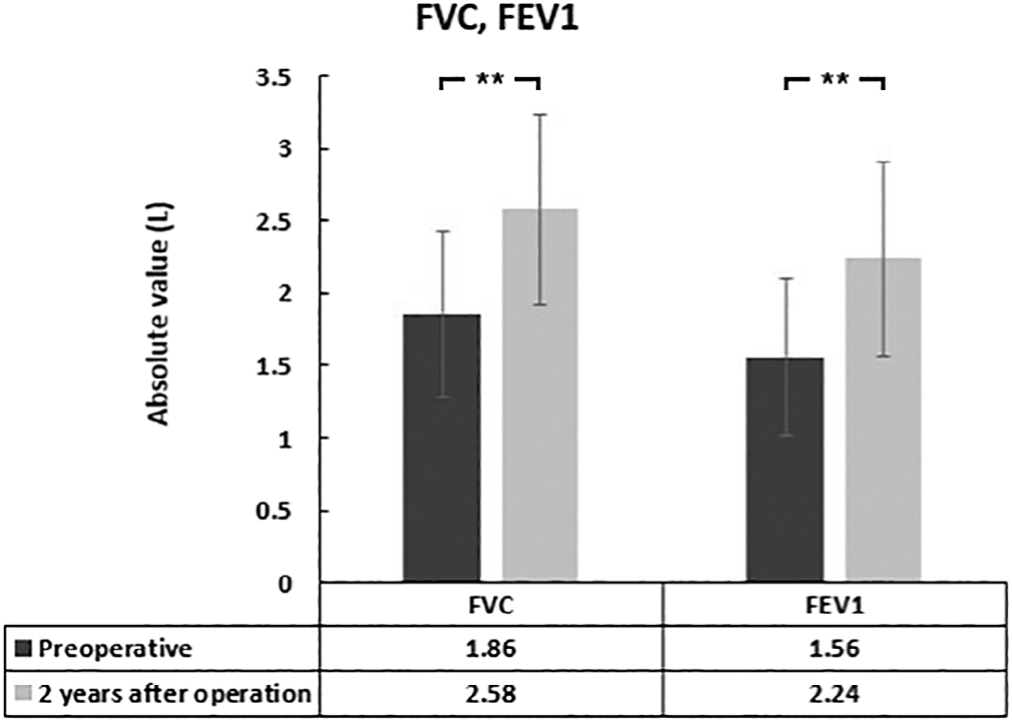
Changes in forced vital capacity (FVC) and forced expiratory volume in 1 s (FEV1) at 2‐year follow‐up. Data represent mean values ± standard deviation for the entire cohort (*n* = 36). Error bars represent standard deviation. **p < 0.01 indicates statistically significant difference between preoperative and 2‐year postoperative values (paired *t*‐test). FEV1, forced expiratory volume in 1 s; FVC, forced vital capacity.

**FIGURE 4:**
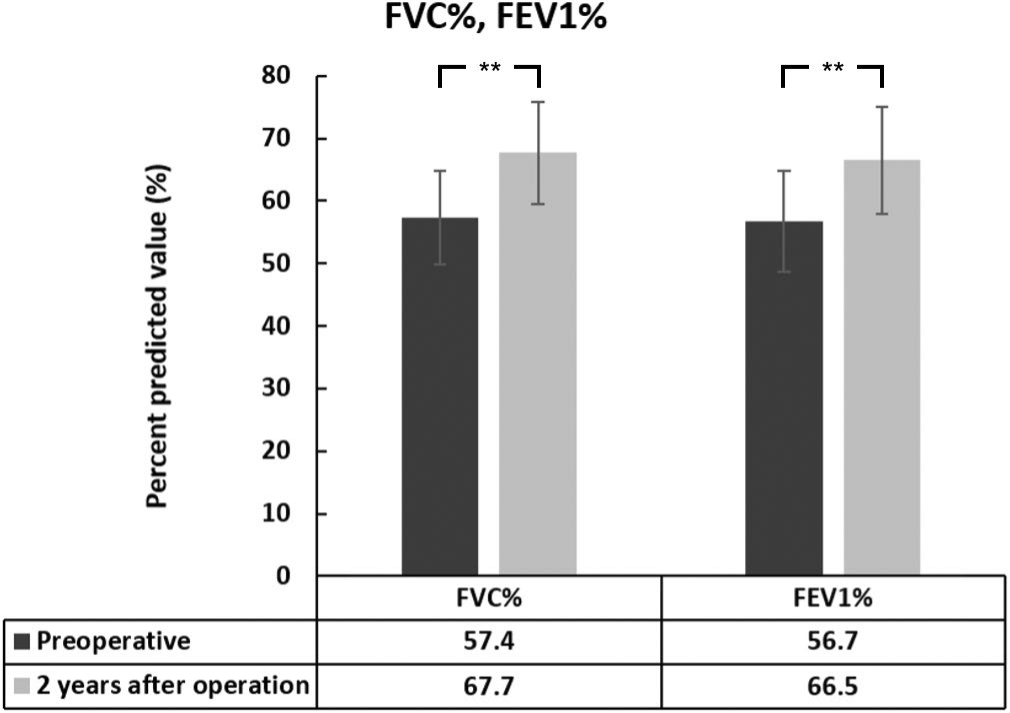
Changes in percent‐predicted forced vital capacity (FVC%) and forced expiratory volume in 1 s (FEV1%) at 2‐year follow‐up. Data represent mean values ± standard deviation for the entire cohort (*n* = 36). Error bars represent standard deviation. **p < 0.01 indicates statistically significant difference between preoperative and 2‐year postoperative values (paired *t*‐test). FEV1%, percent‐predicted forced expiratory volume in 1 s; FVC%, percent‐predicted forced vital capacity.

Preoperative PF results showed RVD (FVC = 1.86 ± 0.57 L, FVC% = 57.4% ± 7.5%; FEV1 = 1.56 ± 0.54 L, and FEV1% = 56.7% ± 8.1%). Before surgery, RVD was present in 97.2% of patients; mild, moderate, and severe RVD accounted for 22.2%, 33.3%, and 41.7% of cases, respectively. At the 2‐year follow‐up, the proportion of RVD was 86.1%, with 19.4% classified as mild, 44.4% as moderate, and 22.2% as severe. After 2 years of ACGB treatment for scoliosis, RVD‐related PF parameters (FVC, FVC%, FEV1, and FEV1%) improved significantly. PF values were improved to FVC = 2.58 ± 0.65 L, FVC% = 67.7% ± 9.7%; FEV1 = 2.24 ± 0.67 L, and FEV1% = 66.5% ± 8.5%. Compared with the preoperative results, FVC, FEV1, FVC%, and FEV1% showed mean increases of 0.72, 0.68 L, 10.3%, and 9.8%, respectively. However, the FEV1/FVC ratio showed no significant difference before surgery and at the 2‐year follow‐up. Representative cases and details of the changes in PF are shown in Figures 1, 2, 3, 4.

### 3.3 Greater TH Improvements and Poorer Preoperative PF Result in Greater Improvements in PF Values After Surgery

All possible variables in this study were analyzed to examine linear relationships with the improvement in PF values. Variables that significantly related to the improvement in any PF value are listed in Table 2. PF value improvements were significantly correlated with age, ΔTH, ΔTD, number of operated thoracic vertebrae, preoperative BMI, type of kyphosis, and preoperative FVC, FEV1%, and FVC%. However, no significant linear correlation was found between the PF value improvements and preoperative scoliosis angle, scoliosis correction rate, kyphosis correction rate, ΔTW, or type of scoliosis.

**TABLE 2:** The linear relationship between improvements in pulmonary function values and possible variables (*r*/*r*_*s*_).

Variables	ΔFVC	ΔFEV1	ΔFVC%	ΔFEV1%
Continuous variables^a^				
Age	−0.318*	0.332*	0.215	0.294*
ΔThoracic height	0.329**	−0.408**	−0.357*	−0.336*
ΔThoracic depth	0.256	0.334*	0.207	0.225
Preoperative FVC	−0.269*	−0.223	−0.296*	−0.214
Preoperative FVC%	−0.359*	−0.215	−0.366*	−0.227*
Preoperative FEV1%	−0.189	−0.225	−0.246	−0.425**
Number of operated thoracic vertebrae	0.325*	0.414*	0.124	0.147
Preoperative body mass index	−0.315*	−0.342*	−0.123	−0.207
Major curve correction rate	−0.106	0.047	−0.084	−0.031
Major curve correction angle	0.076	0.123	0.123	0.021
Categorical variables^b^				
Preoperative type of kyphosis	−0.198	−0.256*	−0.084	−0.043

*Note*: Δ indicates the difference between the results of the 2‐year follow‐up and the preoperative results.

Abbreviations: FEV1, forced expiratory volume in 1 s; FVC, forced vital capacity; FVC%, FEV1%, percent predicted values.

^a^ Two‐tailed Pearson correlation test.

^b^ Two‐tailed Spearman correlation test.

* Correlation was significant at the 0.05 level (2‐sided).

** Correlation was significant at the 0.01 level (2‐sided).

**FIGURE 5:**
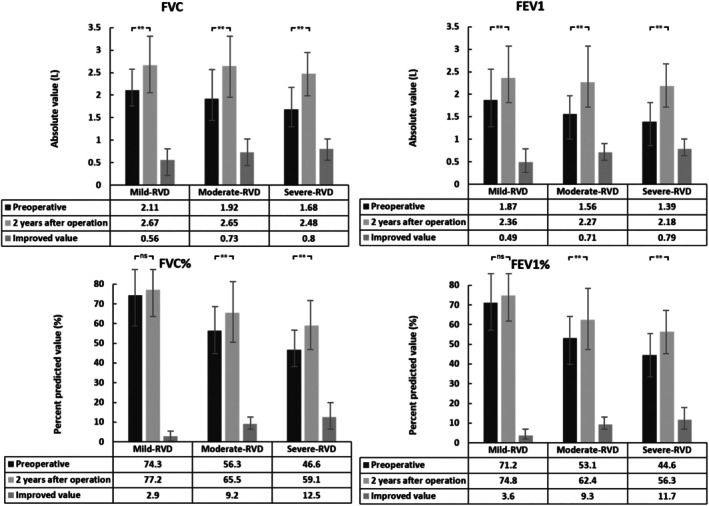
Changes in pulmonary function in patients with different restrictive ventilatory dysfunction (RVD) at 2‐year follow‐up. Patients were divided into three groups based on preoperative FEV1%: Mild‐RVD (FEV1% ≥ 70%, *n* = 9), Moderate‐RVD (50% ≤ FEV1% < 70%, *n* = 15), and Severe‐RVD (FEV1% < 50%, *n* = 26). Error bars represent standard deviation. **p < 0.01 indicates a statistically significant difference between preoperative and 2‐year postoperative values within each RVD group (paired *t*‐test); ns, not significant (*p* > 0.05). FEV1, forced expiratory volume in 1 s; FVC, forced vital capacity; FVC% and FEV1%, percent‐predicted values; RVD, restrictive ventilation dysfunction.

Multiple linear regression analysis further explored the relationship between the improvement in PFT values and all possible impact variables. The details of the variables in the regression equation are shown in Table 3. Variables excluded using the stepwise method in the multiple linear regression analysis are not shown in Table 3. Regression analysis showed that age and ΔTH were significantly correlated with the improvement in FVC and FEV1, and the two variables together explained 31.9% and 36.8% of the improvement in FVC and FEV1, respectively. The preoperative FVC% and ΔTH were significantly correlated with the improvement in FVC%, which together explained 34.1% of the improvement in FVC%. The only variable that was significantly correlated with FEV1% improvement was preoperative FEV1%, which explained 19.2% of the FEV1% improvement. Furthermore, ΔTH positively affected PF value improvements, suggesting that the greater improvement in TH, the greater improvement in postoperative PF values. At the postoperative 2‐year follow‐up, FVC and FEV1 values significantly (*p* < 0.05) increased in the three RVD subgroups, and FVC% and FEV1% values significantly (*p* < 0.05) increased in the moderate and severe RVD groups. Conversely, FVC% and FEV1% only slightly (*p* > 0.05) increased in the mild RVD group (Figure 5). Age, preoperative FVC%, and FEV1% negatively affected PF improvement, indicating that the lower the age at operation, the weaker the preoperative PF and the greater the improvement in PF values at the 2‐year follow‐up.

**TABLE 3:** multiple linear regression analysis between improvements in pulmonary function values and possible variables.

ΔPF values	*R* ^2^	ΔThoracic height		Age		Preoperative FVC%		Preoperative FEV1%	
		*β*	*p*	*β*	*p*	*β*	*p*	*β*	*p*
ΔFVC	0.319	0.261	0.001*	−0.033	0.012*	/	/	/	/
ΔFEV1	0.368	0.244	0.004*	−0.021	0.000*	/	/	/	/
ΔFVC%	0.341	3.607	0.023*	/	/	−0.373	0.001*	/	/
ΔFEV1%	0.192	/	/	/	/	/	/	−0.342	0.000*

*Note*: Δ indicates the difference between the results of the 2‐year follow‐up and the preoperative results. β indicates the β‐coefficient in multiple linear regression analysis.

Abbreviations: FEV1, forced expiratory volume in 1 s; FVC, forced vital capacity; FVC%, FEV1%, percent predicted values.

* Indicates statistical significance at *p* < 0.05.

### 3.4 Complications

All patients successfully underwent one‐stage posterior ACGB surgery, and no revision surgery was performed during follow‐up. The spinal cord monitoring signals of all patients showed no obvious changes during the operation. No pulmonary complications, such as pleural rupture, pulmonary infection, pleural effusion, or respiratory failure, occurred during the perioperative period.

Regarding non‐pulmonary complications, one patient developed superior mesenteric artery syndrome after surgery but was cured by diet adjustment. Two patients experienced delayed wound healing after surgery, but in both cases, wounds healed adequately after conservative treatment for 2–3 weeks. No perioperative neurological complications, pseudarthrosis, implant failure, or deep wound infections were observed in any patient.

## 4 Discussion

ACGB is a one‐stage, three‐rod surgical strategy for severe scoliosis, which requires only low‐grade osteotomy (Grade I or II) but can achieve a reasonable corrective effect. However, the effect of ACGB on PF in patients with severe thoracic scoliosis remains unknown. In our cohort of scoliosis patients treated with ACGB, the RVD‐related PF values (FVC, FVC%, FEV1, and FEV1%) significantly improved 2 years after the operation. Age, ΔTH, preoperative FVC%, and preoperative FEV1% may be related to the improvement in PF.

### 4.1 Correction of Scoliosis and Pulmonary Function

Several alternative surgical methods can achieve good corrective effects for mild‐to‐moderate scoliosis; however, the effects of different surgical methods on postoperative PF are very different [13, 14]. Surgical procedures that have a direct effect on the thoracic cage and lungs, such as anterior release or vertical expandable prosthetic titanium rib (VEBTR), may cause pulmonary complications and reduce postoperative medium‐term PF [13, 14, 15, 16]. However, Kim et al. [17] found that the absolute and percent‐predicted PF values of patients with adolescent idiopathic scoliosis (AIS) who underwent posterior segmental spinal fusion alone significantly improved during the 2‐year follow‐up.

The choice of surgical method for severe scoliosis has diversified. High‐grade osteotomies, such as pedicle subtraction osteotomy (PSO) or vertebral column resection (VCR), are traditionally recommended for severe scoliosis [18]. VCR has been reported to correct severe scoliosis by 51%–67% in numerous studies [19, 20, 21]. Despite effective correction of scoliosis, whether PF can be improved after surgery remains unclear [6, 19]. Zhang et al. [19] conducted a retrospective study of 53 patients with severe scoliosis who received VCR and found that PF was significantly improved 2 years after VCR (FVC, 1.59–1.95 L; FEV1, 1.44–1.77 L; FVC%, 49%–56%; FEV1%, 52%–57%). However, Bumpass et al. [6] also followed up on patients with severe scoliosis who received VCR and found only a small improvement in the absolute values of FVC and FEV1 in pediatric patients (FVC, 2.10–2.43 L; FEV1, 1.71–1.98 L), while no improvement in PF was found in adult patients. The difference in the improvement of PF after VCR may be because VCR uses compression to close the spinal osteotomy and shorten the TH, while ACGB only uses low‐grade osteotomy and distracts the spine, which increases the TH. Besides, Lenke et al. reported that 59% of patients with severe scoliosis who underwent VCR experienced perioperative complications, and Gupta et al. similarly documented a 58% complication rate in severe pediatric spinal deformity with VCR, which also will affect postoperative PF [21, 22]. In contrast, our ACGB approach utilizing low‐grade osteotomies throughout the fusion range demonstrated a complication rate (8.3%), with no major complications observed, combined with reasonable deformity correction (51.5%). Anterior release combined with posterior spinal fusion achieved a good correction rate of 49%–75% for severe scoliosis, with a complication rate of 9.6%–26.8% [20, 23, 24, 25]. Although a good correction rate has been achieved, due to thoracic surgery and the high incidence of pulmonary complications, the improvement in postoperative PF was not significant [26]. Currently, halo traction is widely used to treat severe scoliosis [27]. Previous studies have shown corrective rates of 46%–53% for preoperative halo‐pelvic traction and 50.8%–58.3% for intraoperative skull‐femoral traction, with a traction‐related complication rate of 16%–28% [7, 23, 28, 29]. Many researchers have further confirmed that preoperative halo traction not only achieves a certain corrective effect but also improves PF, especially by increasing FVC% and FEV1% [23, 30].

The use of a three‐rod surgical strategy for the treatment of severe scoliosis can be traced back to De Giorgi et al. [31] in the 1990s when Cotrel–Dubousset instrumentation was applied to correct severe scoliosis. Currently, a strong corrective force can be obtained in the apical region using pedicle screws alone, which makes it possible for ACGB surgical strategies to treat severe scoliosis [9]. While achieving a reasonable correction rate (51.5%) and a very low incidence of complications, our study suggests that ACGB can improve PF in patients with severe scoliosis, 2 years after surgery. We propose several reasons to explain this: first, high‐grade osteotomy was not performed, so pulmonary complications, such as pleural rupture, were relatively low; second, ACGB was a simple one‐stage posterior operation, which would not damage the ribs and lungs from the anterior position; and third, the relatively straight short rod in the apical region was segmentally distracted, which can provide more corrective force than long curved rods, indirectly increase the TH, and improve the thoracic volume.

### 4.2 Possible Factors Affecting Improvement in PF With the ACGB Surgical Strategy

In our study, multiple linear regression showed that ΔTH, age, and preoperative FVC% and FEV1% were related to the improvement in PF. Among these factors, ΔTH was related to the improvements of FVC, FEV1, and FVC%, which may suggest that ΔTH was an important factor in PF improvement. The mean increase in TH after the operation was 2.8 cm (*p* < 0.05), whereas the TW and TD did not significantly change. This selective improvement of thoracic height without transverse or anteroposterior narrowing represents an important characteristic of the ACGB surgical strategy and has significant implications for PF recovery. The significant increase in TH levels may be because only low‐grade osteotomy was performed during ACGB. High‐grade osteotomy involves partial or complete removal of the vertebral body and adjacent discs, and compression is required to close the area of the osteotomy, which not only improves the correction rate but also shortens the TH in disguise [32]. In contrast, the ACGB surgical strategy does not shorten the anterior or middle column of the thoracic vertebrae and effectively increases the TH by means of segmental distraction of the short rod in the apical region, such that the TH can be significantly increased. Consequently, the ACGB strategy could achieve PF rebuilt through improving TH while preserving TW and TD. Many studies have also shown that TH is closely associated with PF in patients with scoliosis [33]. Karol et al. [34] and Pehrsson et al. [35] found that insufficient TH (T1‐T12, < 18 cm) could lead to RVD. Our study showed that surgical improvement in TH in patients with scoliosis helps improve postoperative PF.

Additionally, our analysis found no significant correlation between major curve correction and PF improvements, which is consistent with findings by Zhang et al. [19] in severe spinal deformity patients. This suggests that thoracic volume instead of curve correction may be the key determinant of PF recovery. This could explain why high‐grade osteotomies show limited PF improvement despite better correction rates.

Age at operation was negatively correlated with the absolute values of FVC and FEV1 improvements; however, no linear correlation was found between the percentage predicted values (FVC% and FEV1%), which may be because the predicted percentage values were normalized to age. This result suggests that the absolute PF improvement values at the 2‐year follow‐up were relatively high in younger patients, but improvements in percent predicted values were not significant in younger patients.

The preoperative FVC% and FEV1% were negatively correlated with PF improvement for the percent predicted values. Further, the difference in PF improvement values among the subgroups of RVD also suggested that patients with poorer preoperative PF may show greater postoperative improvement. Byun et al. [36] also found no significant difference in PF values in patients 15 years after posterior spinal fusion, but a significant improvement in PF values was observed in patients with severe PF prior to surgery.

### 4.3 Limitations and Strengths

This study has several limitations. First, the sample size was relatively small with heterogeneous etiologies, which may limit the generalizability of our findings. To minimize the potential impact of diverse etiologies, we applied strict inclusion and exclusion criteria, excluding patients with diseases that could directly affect PF. Etiological subgroup analysis was also conducted to address this concern. Future multicenter studies with larger, more homogeneous patient populations would be valuable to further validate our findings. Second, the follow‐up period was limited to 2 years. PF changes after scoliosis surgery represent a long‐term process, and previous studies have shown that PF may continue to evolve for many years postoperatively. Longer follow‐up would assist in assessing the sustained effects of the ACGB strategy on PF in severe scoliosis patients. Extended follow‐up studies are planned to address this important question in the future.

Despite these limitations, our study has several notable strengths. First and most importantly, we introduced and evaluated the ACGB surgical strategy, which improves thoracic height and elongates spinal length through segmental distraction rather than compression, fundamentally differentiating it from traditional high‐grade osteotomies that shorten the spine. This study systematically assessed the impact of this approach on both PF and spinal parameters in severe scoliosis patients. Second, our comprehensive analysis incorporated detailed three‐dimensional thoracic measurements, revealing that increased thoracic height is the key mechanism for PF improvement. Third, despite heterogeneous etiologies, our subgroup analysis demonstrated consistent improvements across different scoliosis types, enhancing the generalizability of our findings. Finally, the combination of reasonable deformity correction (51.5%) with significant PF improvement and low complication rates (8.3%) demonstrates that ACGB offers a safer and more effective alternative to high‐risk procedures such as vertebral column resection for treating severe scoliosis.

## 5 Conclusion

The ACGB surgical strategy can significantly improve postoperative PF in patients with severe scoliosis at 2‐year follow‐up. The increased TH may be crucial for improving PF values, while patients with poorer preoperative PF may show greater postoperative PF improvement.

## Author Contributions

**Junduo Zhao:** visualization, investigation, writing – review and editing. **Yang Jiao:** data curation, writing – original draft, writing – review and editing. **Yizhen Huang:** software, data analysis. **Heng Sun:** software, validation, data analysis. **Haoyu Cai:** investigation, data collection. **Haojie Chen:** investigation, data collection. **Jianxiong Shen:** conceptualization, methodology, supervision, writing – review and editing.

## Funding

This work was supported by the National Natural Science Foundation of China, 82230083; the Postdoctoral Fellowship Program of China Postdoctoral Science Foundation, GZC20251383.

## Disclosure

All authors listed meet the authorship criteria according to the latest guidelines of the International Committee of Medical Journal Editors (ICMJE). All authors are in agreement with the content of the manuscript and have approved the final version for submission.

## Ethics Statement

Ethics approval and consent to participate: All patients and legal guardians provided written informed consent. This study was approved by the institutional ethics committee (reference no: S‐K1564‐20150626).

## Consent

All patients consented to the publication of their data.

## Conflicts of Interest

The authors declare no Conflicts of Interest.

## Data Availability

The data that support the findings of this study are available from the corresponding author upon reasonable request.
